# Study on Vibration and Acoustic Protection of Indium EUV Filters for Space Payloads

**DOI:** 10.3390/mi17060649

**Published:** 2026-05-25

**Authors:** Shilei Mao, Bo Chen, Quanfeng Guo, Chunyang Han, Lingping He, Hongji Zhang

**Affiliations:** 1 Changchun Institute of Optics, Fine Mechanics and Physics, Chinese Academy of Sciences, Changchun 130033, China; maosl@ciomp.ac.cn (S.M.);; 2 University of Chinese Academy of Sciences, Beijing 100049, China

**Keywords:** indium extreme ultraviolet (EUV) filters, EUV camera, vibration isolation, acoustic mitigation, space payload

## Abstract

Extreme ultraviolet (EUV) thin-film filters are key optical components in space-based EUV imaging, employed to reject out-of-band visible and ultraviolet radiation. Currently, aluminum (Al) and zirconium (Zr) EUV filters are predominantly used due to their superior mechanical strength and stability. In contrast, indium (In) EUV filters had not been successfully deployed in spaceborne EUV cameras prior to this work. Their inherent fragility makes them highly susceptible to vibration and acoustic loads during the launch phase, ultimately resulting in structural failure. This study presents a comprehensive investigation into the structural protection strategies for indium EUV filters in space applications. Through systematic analysis of the photoenergy requirements and the mechanical characteristics of the indium filters, a robust filter assembly was developed, and vibration isolation as well as acoustic mitigation designs were implemented for the assembly. Finite element simulations and environmental tests confirmed that the indium filters can withstand vibration and acoustic loads. This technology has been successfully implemented in the 83.4 nm channel of the Extreme Ultraviolet Camera (EUVC) onboard the Queqiao-2 relay satellite for the Chang’E-7 mission. Subsequent in-orbit tests validated the structural integrity of the indium filters, providing a valuable technical reference for their successful application in future spaceborne EUV cameras.

## 1. Introduction

Extreme ultraviolet (EUV) radiation, spanning wavelengths from 10 to 120 nm [1], offers invaluable insights into solar physics and Sun–Earth system dynamics [2,3,4,5]. Disturbances in the geospace environment, driven by solar activity, often appear as magnetic storms, substorms, and other space weather phenomena, posing significant threats to satellite operations, telecommunications, and navigation systems. Consequently, imaging in the EUV bands enables comprehensive monitoring of solar activity and magnetospheric dynamics, providing critical data for space weather modeling and prediction.

The Earth’s atmosphere is almost entirely opaque to solar EUV radiation due to strong absorption by ozone, atomic oxygen, and nitrogen species, thereby restricting EUV observations exclusively to space-based platforms. Despite its critical importance, spaceborne EUV instrumentation faces formidable challenges, including intrinsically weak photon fluxes, the lack of transmissive optical materials, limited mirror reflectivity (typically <50% in the EUV bands), stringent surface roughness requirements (<0.5 nm RMS), and difficulties in out-of-band rejection. A paradigm shift occurred in the 1980s, driven by concurrent advancements in three key technologies: (1) ultra-precision surface finishing, (2) multilayer interference coatings [6], and (3) thin-film filter deposition. The integration of multilayer mirrors with EUV thin-film filters paved the way for breakthrough achievements in narrowband space EUV imaging.

Metal thin-film filters exhibit wavelength-dependent spectral transmission characteristics determined by their material composition and thickness. Through optimization, these filters can transmit specific EUV radiation while effectively suppressing out-of-band radiation. However, the requirement for the sub-200 nm thickness makes the metal films structurally fragile, necessitating their bonding to perforated support grids for mechanical reinforcement. This structure significantly enhances mechanical stability without compromising optical performance. Nevertheless, the survival of these filters during rocket launch remains a critical technological challenge, as extreme vibration and intense acoustic loads pose substantial risks of structural failure. Ensuring the survivability of metal thin-film filters during the launch phase is a key factor for the successful operation of space-based EUV imaging systems [7,8,9,10,11].

The 1995 SOHO mission [12,13], featuring the Extreme ultraviolet Imaging Telescope (EIT), pioneered space-based narrowband solar imaging at four EUV wavelengths (17.1, 19.5, 28.4, and 30.4 nm) [14,15]. The EIT employed a unique optical design with quadrant-specific multilayer mirrors and entrance Al filters, utilizing a protective door to shield the filters from launch vibrations. This successful architecture set the precedent for subsequent NASA missions like TRACE, STEREO, and SDO [16,17,18,19,20]. These satellites deployed similar EUV imagers that retained key features: entrance Al filters and Zr filters for blocking non-EUV radiation, and protective doors to ensure structural survival during launch.

Launched in 2000, the Extreme Ultraviolet Imager (EUVI) aboard NASA’s Imager for Magnetopause-to-Aurora Global Exploration (IMAGE) mission achieved breakthrough imaging of the Earth’s plasmasphere. The EUVI instrument primarily comprised entrance Al thin-film filters, a spherical mirror, and a photon-counting imaging detector [21]. The synergistic combination of Al filters and a multilayer mirror facilitated effective out-of-band rejection, while the photon-counting detector enabled the observation of weak signals. Additionally, a protective door was integrated at the optical entrance to shield the delicate Al filters from launch-induced damage.

In 2008, Japan launched the SELENE satellite, which carried an EUV camera featuring a single-mirror optical system and a thin-film filter positioned at the pupil plane. The filter was segmented into two regions: the upper section utilized an Al/C thin-film filter for 30.4 nm imaging, while the lower section employed an indium thin-film filter for 83.4 nm imaging. A protective door was also designed at the entrance aperture to safeguard the filters. However, structural failure of the indium filter precluded the full completion of the scheduled observation mission. Nevertheless, this camera provided valuable experience for the future space application of indium thin-film filters. This failure directly led subsequent EUV space cameras to mostly abandon indium EUV filters.

In 2013, China’s Chang’e-3 lander successfully landed on the lunar surface, where its onboard EUV camera achieved the first EUV imaging of the Earth’s plasmasphere from the Moon at the 30.4 nm band [22,23]. The instrument employed a single spherical reflector system. An Al thin-film filter, combined with a multilayer mirror at the optical entrance, allowed imaging at 30.4 nm while effectively rejecting out-of-band radiation. Additionally, a protective door was integrated at the aperture to protect the thin-film filter during launch.

The Extreme Ultraviolet Camera (EUVC) onboard the Queqiao-2 relay satellite for the Chang’e-7 mission [24,25], featuring an opto-mechanical architecture similar to that of the Chang’e-3 EUV imager, operated in lunar orbit to perform Earth imaging at the 30.4 nm and 83.4 nm dual bands. The 83.4 nm channel uses 200 nm thick indium filters [26,27]. The 83.4 nm extreme ultraviolet camera incorporating the indium filters is required to withstand a random vibration load of 8.5 g_rms_ and an acoustic load of 142 dB.

Currently, indium EUV filters face significant challenges due to their inherent fragility and the lack of successful on-orbit experience. This study proposes a reliable design and protection scheme for indium EUV filters, which has been successfully applied to the EUVC onboard the Queqiao-2 relay satellite for the Chang’e-7 mission. This achievement fills the gap in successful on-orbit EUV imaging observations using indium EUV filters for space applications. This proposal includes two key innovations:

(1) Structural design: Design of the indium filter assembly structure;
(2) Environmental adaptability: Vibration isolation and acoustic mitigation designs.

## 2. Structural and Protection Designs of Indium Extreme Ultraviolet (EUV) Filters

### 2.1. Mechanical Structure Design

Indium EUV filters are susceptible to breakage or even falling off during launch-induced mechanical and acoustic stresses. The debris from indium EUV filters can cause the photon-counting detector to malfunction under high voltage when the indium EUV filters are broken. Therefore, the filters are placed at the pupil of the camera.

A 200 nm thick indium film is plated onto a metal support grid to enhance the thin-film filters. The shape of the filters can be either circular or sector-shaped, with sector-shaped filters having a clear advantage in terms of aperture area, whereas circular filters perform better in terms of mechanical properties. Based on a comprehensive evaluation, the circular filter configuration is finally selected.

**Figure 1:**
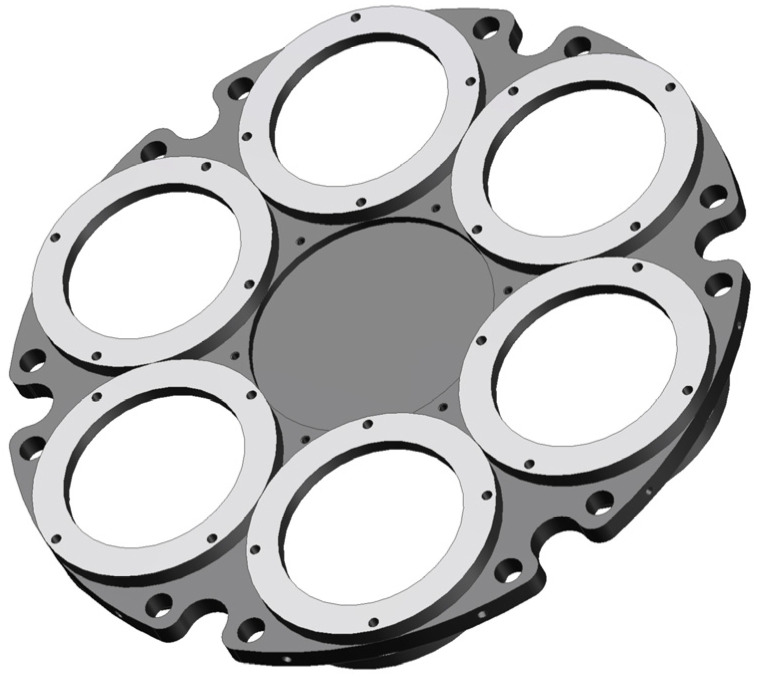
The indium EUV filter assembly.

All indium thin-film filters are uniformly bonded to the filter mount by silicone rubber without using any fasteners in order to fully eliminate mounting stress. The structure is shown in Figure 1. Aluminum alloy is chosen as the material for both the filter frame and the filter mount, which effectively reduces thermal stresses under temperature loads. The overall diameter of the indium EUV filter assembly is 160 mm, and the clear aperture of each indium thin-film filter is 40 mm.

The indium EUV filter assembly is installed at the entry pupil of the 83.4 nm camera, and divides the camera into two acoustic cavities: the upper acoustic cavity near the door side and the lower acoustic cavity near the mirror side. The upper acoustic cavity mainly consists of two parts, the door and the front barrel, with the door used to protect the filters from being damaged during the rocket launch. The lower acoustic cavity mainly consists of two parts, the mid-barrel and the rear barrel. The 83.4 nm camera features overall dimensions of 450 × 300 × 260 mm, with a 220 mm diameter primary mirror and a focal length of 147.45 mm.

### 2.2. Vibration Isolation Design

By introducing metal support grids and adopting circular geometries, the mechanical properties of thin-film metal filters have been improved. Al and Zr EUV filters can withstand launch-induced vibration loads without additional isolation. In contrast, indium EUV filters remain susceptible to damage during rocket launches and require vibration isolation to ensure structural integrity. Empirical data indicate that indium filters fracture when the assembly’s acceleration response reaches 20 g_rms_ in random vibration tests. Consequently, a vibration isolation system is essential to limit the response to below 15 g_rms_, thereby ensuring an adequate safety margin.

There are two categories of vibration isolation: active vibration isolation and passive vibration isolation. Active vibration isolation counteracts external vibrations by generating opposing forces or vibrations, and typically incorporates power sources, controllers, and sensors. Consequently, it has a more complex structure and control scheme, with stricter constraints on mass, volume, control, and power consumption, making it unsuitable for the EUVC in this study. Passive vibration isolation achieves isolation by dissipating vibrational energy through damping materials. It offers a simple structure, high reliability, and ease of implementation. Therefore, passive vibration isolation is employed for the filter assembly in the EUVC.

According to the principle of vibration isolation, when the frequency ratio is below $\sqrt{2}$, the vibration isolator amplifies vibration transmission. In particular, a frequency ratio of 1 leads to resonance, and the damping ratio acts to reduce the resonant amplification. When the frequency ratio equals $\sqrt{2}$, the vibration transmissibility is 1 regardless of the damping ratio. The vibration isolation effect becomes significant when the frequency ratio exceeds $\sqrt{2}$. Therefore, when selecting a vibration isolator, the stiffness should be reduced as much as possible to achieve a broad isolation region, and the damping should be increased to suppress amplification in the resonance region.

**Figure 2:**
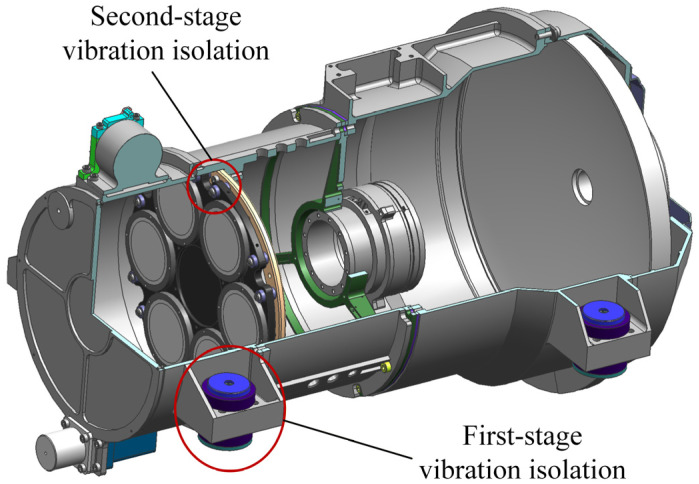
Two-stage vibration isolation system in EUVC.

To further enhance the vibration isolation performance, a two-stage vibration isolation system is employed. Four rubber dampers are positioned between the camera and the mounting base as the first-stage isolation, and six small rubber dampers are arranged between each filter mount and the filter mounting ring as the second-stage isolation. The configuration of the vibration isolation system is shown in Figure 2.

The selection of rubber dampers for the two-stage vibration isolation system is based on the following principles:

(1) Reduce the stiffness of the first-stage dampers so that the natural frequency of the 83.4 nm camera assembly is close to 50 Hz, while ensuring it remains above 50 Hz;
(2) Increase the damping of the first-stage dampers to suppress amplification at the resonance point;
(3) Increase the stiffness of the second-stage dampers so that the natural frequency of the filter assembly exceeds 200 Hz, thereby achieving frequency detuning between the two stages and mitigating resonance.

**Figure 3:**
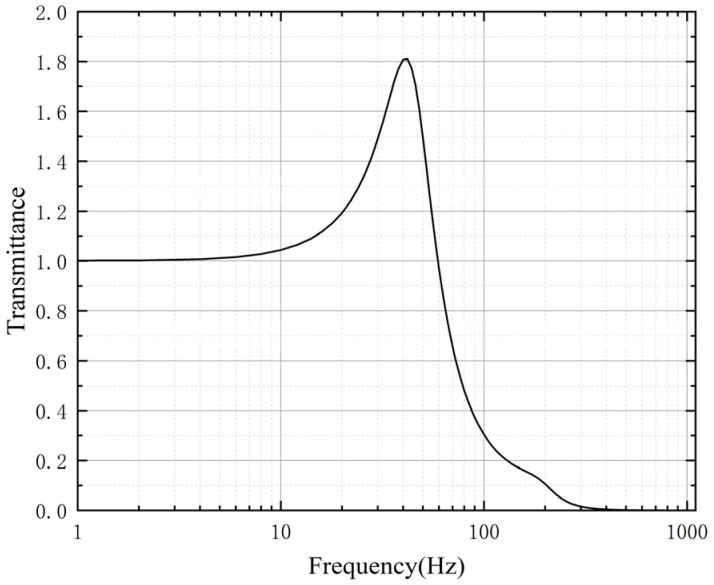
Transmittance curve of the two-stage vibration isolation system.

The transmittance curve of the finalized two-stage vibration isolation system, shown in Figure 3, indicates that the system can effectively reduce high-frequency responses above 60 Hz. Additionally, it provides effective suppression of resonant responses in the 10–60 Hz range, resulting in a resonance amplification factor of less than 1.8.

### 2.3. Acoustic Mitigation Design

In previous studies, Al and Zr EUV filters were protected against acoustic noise using thick protective doors and barrels, which resulted in considerable mass penalties. Compared with Al and Zr EUV filters, the indium EUV filters are more sensitive to acoustic loads and require an optimized acoustic mitigation design under severe mass constraints. Previous test results have shown that indium EUV filters fail when the sound pressure level (SPL) near the filter assembly reaches 130 dB during acoustic tests. Therefore, the SPL near the filter assembly should be controlled below 125 dB to provide an adequate safety margin.

In the aerospace field, acoustic loads primarily originate from exhaust and aerodynamic noise generated during rocket liftoff, which impinges on the fairing surface and may affect the payloads. Current noise mitigation strategies include: (1) source-level suppression; (2) path-level attenuation; and (3) localized protection of noise-sensitive components using either porous sound-absorbing materials for absorption or dense sound-insulating materials for isolation. For the noise suppression of the indium EUV filters in this study, only the third strategy is considered. Porous sound-absorbing materials demand extra installation space and are difficult to implement. Therefore, a combined scheme of sound insulation and sealing measures is ultimately employed.

A solid medium is placed between the sound source and the target. When incident sound waves impinge on the medium, part of the energy is transmitted through the medium, part is reflected by the medium, and part diffracts around the edges of the medium toward the target. If the medium is infinitely large or fully encloses the target, only sound transmitted through the medium can reach the target, which significantly reduces the sound pressure level (SPL) at the target surface. This is the fundamental principle of sound insulation.

According to published papers, ignoring the incidence angle, the approximate formula for the sound transmission loss (STL) of a single-layer sound-insulating medium is as follows [28].

(1) R=20lgf+20lgm−42,

where *R* (dB) represents STL, *f* (Hz) is the frequency of the incident sound, and *m* (kg/m^2^) is the areal density of the sound insulating medium.

According to the sound insulation principle, the structure of the upper cavity needs to be optimized since it is the weak region for sound insulation, and the areal density of the door is particularly critical. First, the door is designed to be sealed, and then the areal density of the upper acoustic cavity structure is increased. However, the mass budget of the EUVC prevents the areal density of the upper acoustic cavity from being increased indefinitely, and the increased areal density of the door also imposes higher requirements on the drive motor. Therefore, the optimized design should maximize the STL of the upper acoustic cavity while satisfying these constraints.

**Figure 4:**
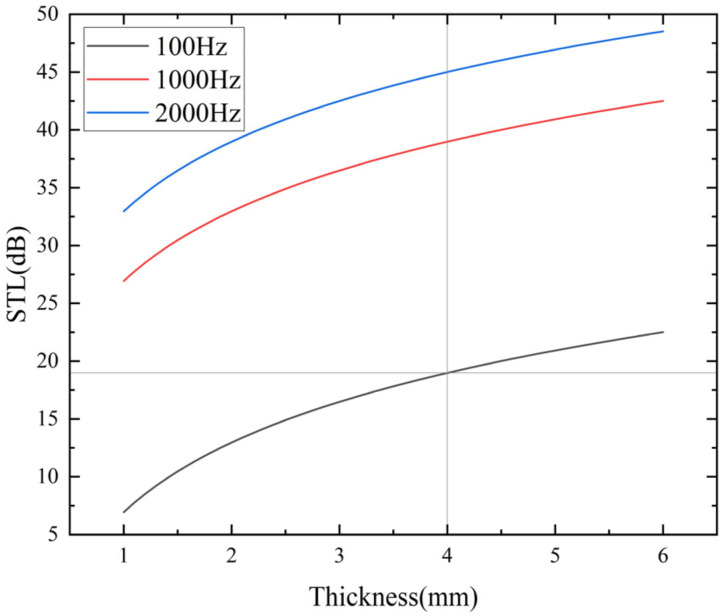
Sound transmission loss (STL) of aluminum alloy structures with different thicknesses at various incident sound frequencies.

The materials used for the upper acoustic cavity structures are aluminum alloys, which feature high specific stiffness and good processability. During rocket launch, the EUVC must withstand an acoustic load of 142 dB SPL, and the SPL around the filter assembly must be kept below 125 dB to ensure the filters remain intact, requiring the STL to be more than 17 dB. Based on Equation (1), the STL of aluminum alloy structures with different thicknesses at various incident sound frequencies is calculated, as listed in Figure 4.

The data in Figure 4 demonstrate a nonlinear relationship between thickness and STL. Increasing the thickness below 4 mm yields a significant improvement in STL, achieving 19 dB attenuation across the three frequency bands. Beyond this threshold, the increase in STL becomes gradual, whereas the increase in mass is more pronounced. The sound insulation structure is designed using a 4 mm thick aluminum alloy, which can achieve an STL of 19 dB in the upper acoustic cavity, verifying the feasibility of the structural sound insulation measure.

The total masses of the door and front barrel with different wall thicknesses are calculated separately and listed in Table 1. Constrained by the driving motor and the total mass budget of the EUVC, the wall thickness of the door should not exceed 5.0 mm, and the total mass should be limited to 500 g. By analyzing the mass and STL corresponding to different thicknesses, a 4 mm thick door and a 2 mm thick front barrel are finally selected.

## 3. Analysis

### 3.1. Dynamic Response Analysis

**Figure 5:**
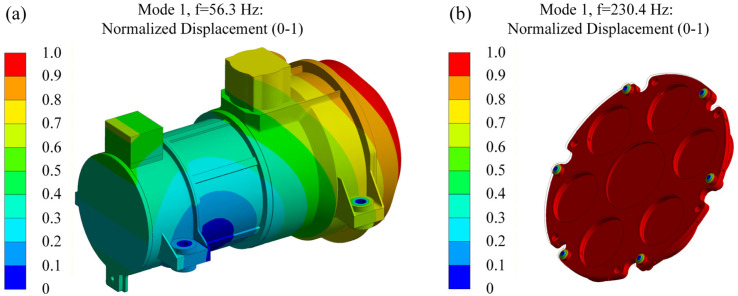
Mode shapes. (**a**) 83.4 nm camera. (**b**) Filter assembly.

To investigate the dynamic characteristics of the 83.4 nm camera and filter assembly and verify the effectiveness of its vibration isolation design, modal analysis was carried out using the finite element method (FEM). The natural frequencies and mode shapes are summarized in Table 2 and Figure 5, respectively.

The results demonstrate that the natural frequency of the 83.4 nm camera is 56 Hz, exceeding 50 Hz and thus satisfying the design requirements. Meanwhile, the natural frequency of the filter assembly is 230.4 Hz, ensuring the frequency detuning of the two-stage vibration isolation system.

### 3.2. Random Vibration Response Analysis

Random vibration response analysis was performed on the 83.4 nm camera and filter assembly. The random vibration requirements are listed in Table 3, with a loading duration of 1 min. The acceleration response results of the 83.4 nm camera and filter assembly are presented in Table 4, where the response values are expressed as root mean square (RMS).

The results indicate that, with the vibration isolation system, the response of the 83.4 nm camera is reduced in all three directions. For the filter assembly, the response is decreased in all directions except the X-direction. However, the acceleration response in all directions remains below 15 g_rms_. Therefore, the indium EUV filters are intact.

### 3.3. Acoustic Analysis

**Figure 6:**
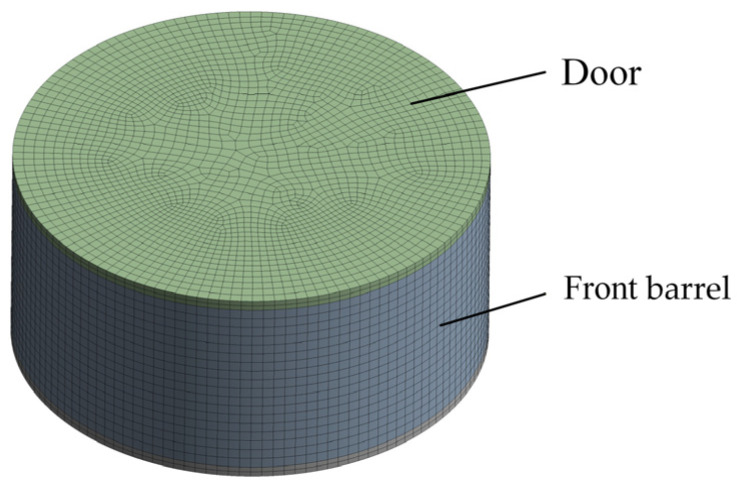
Finite element analysis model.

The sound insulation performance of the upper acoustic cavity was analyzed using the finite element method. A coupled structural–acoustic finite element model was established. Subsequently, external acoustic excitation was applied to the structure. The finite element model is shown in Figure 6.

The finite element model consists of two components: the door and the front barrel, with a sealed boundary condition between them. The acoustic test requirements for the EUVC are listed in Table 5. Given the challenges of high-frequency simulation using the finite element method, and since most of the energy of the external acoustic excitation is concentrated below 2000 Hz while sound insulation performance is more pronounced in the high-frequency band, the excitation frequency range employed in this analysis was set to 20–2000 Hz.

**Figure 7:**
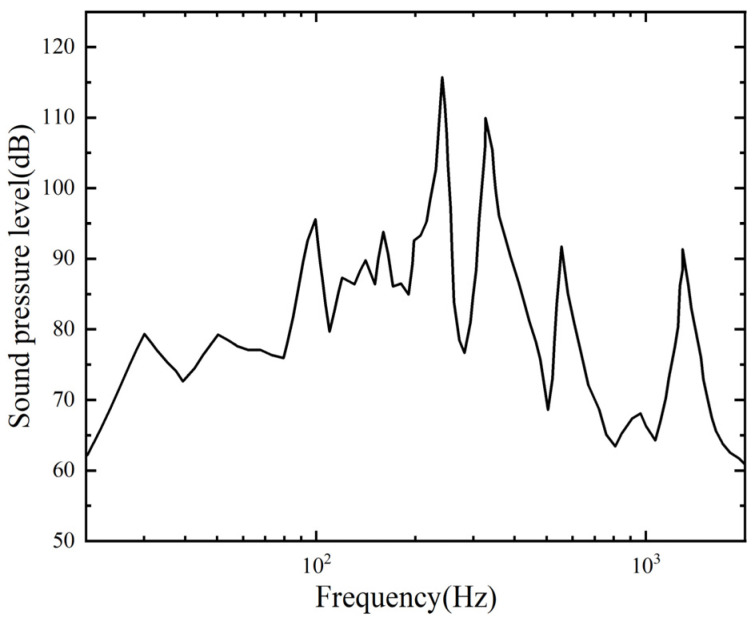
Sound pressure spectrum of the upper acoustic cavity.

The analysis results show that the STL at the filter assembly location within the upper acoustic cavity is 24 dB, exceeding 17 dB and thus meeting the STL requirement. The sound pressure spectrum of the upper acoustic cavity is shown in Figure 7.

## 4. Tests

### 4.1. Vibration Tests

**Figure 8:**
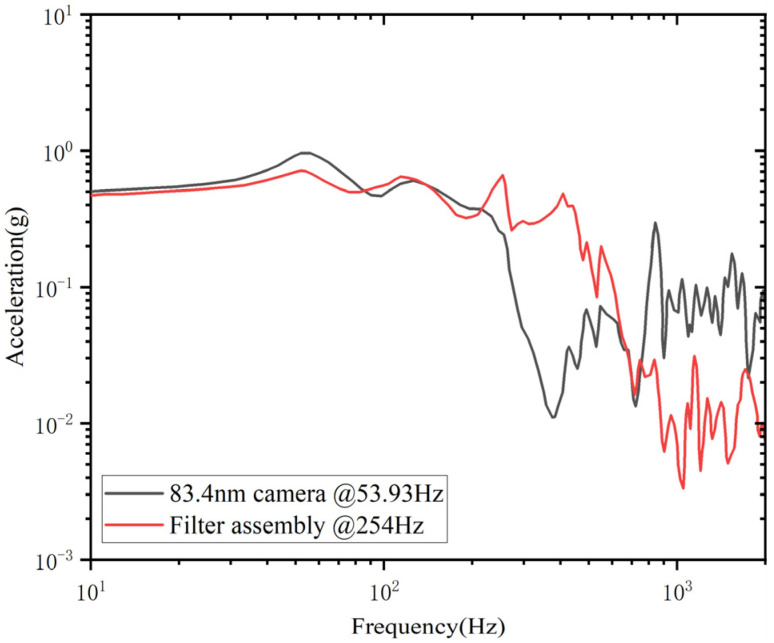
Frequency response curves of EUVC.

The EUVC was mounted on a vibration shaker for experimental testing. A sine sweep vibration test was performed, and the frequency response curves are shown in Figure 8. The results indicate that the natural frequencies of the 83.4 nm camera and filter assembly are 53.9 Hz and 254.0 Hz, respectively, which satisfy the requirement that the natural frequency should exceed 50 Hz and ensure frequency detuning. The test results are in good agreement with the analysis results, thereby validating the vibration isolation design.

Subsequently, random vibration tests were conducted. The test input specifications are listed in Table 3, and the test results are summarized in Table 6. The results demonstrate that, with the two-stage vibration isolation system, the random vibration responses of the 83.4 nm camera in all three directions are lower than the input excitation levels, and those of the filter assembly in all three directions are below 15 g_rms_. Post-test inspection confirmed that the indium EUV filters remained intact.

### 4.2. Acoustic Tests

**Figure 9:**
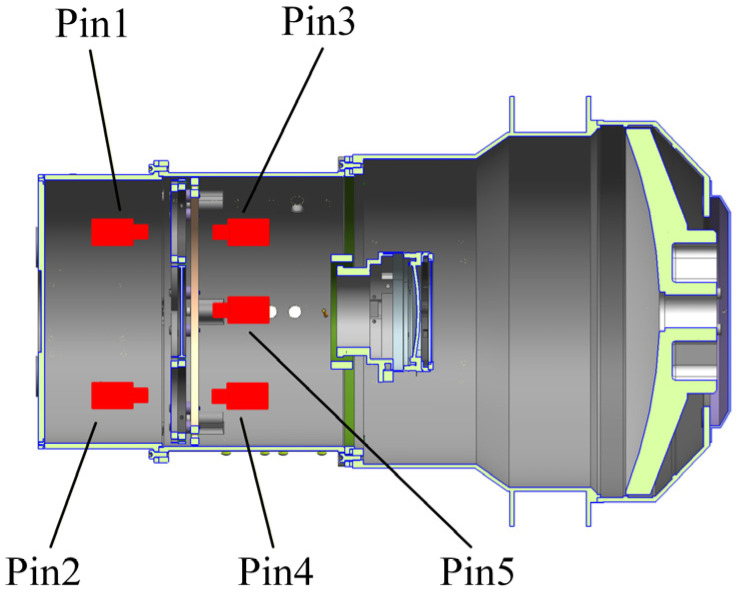
Microphones layout in EUVC.

The EUVC was placed in a reverberation chamber for acoustic tests, with the test specifications listed in Table 5. Five microphones (Pin1–Pin5) were installed in the upper and lower acoustic cavities, and an additional microphone (Pin0) was positioned at the center of the reverberation chamber to monitor the external acoustic excitation, as shown in Figure 9.

**Figure 10:**
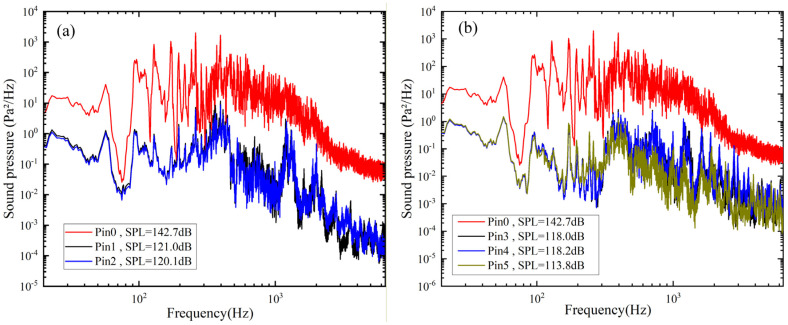
Sound pressure spectra. (**a**) External excitation and upper acoustic cavity; (**b**) External excitation and lower acoustic cavity.

The sound pressure spectra of the external acoustic excitation and the upper/lower acoustic cavities are presented in Figure 10. The results show that under an external excitation of 142 dB SPL, the SPL values are 120.5 dB in the upper acoustic cavity and 116.7 dB in the lower acoustic cavity, both of which are below 125 dB. These results validate the acoustic mitigation design. Post-test inspections confirmed that the indium EUV filters remained intact.

## 5. Conclusions

This study has been carried out to implement the mechanical structure, vibration isolation, and acoustic mitigation designs of the indium EUV filters used in the EUVC onboard the Queqiao-2 relay satellite for the Chang’e-7 mission. The effectiveness of these designs was validated through finite element simulations and experimental tests.

Vibration test results indicated that the two-stage vibration isolation system successfully restricted the acceleration response of the filter assembly to below 15 g_rms_ under random vibration excitation, thereby effectively ensuring the structural integrity of the indium EUV filters. Acoustic tests demonstrated that, with the optimized acoustic mitigation configuration, the STL of the upper acoustic cavity reached 22.2 dB, exceeding the required 17 dB and providing a sufficient safety margin for the filter.

These designs have been applied to the EUVC onboard the Queqiao-2 relay satellite for the Chang’e-7 mission, which was launched in 2024 and currently operates normally in orbit.

## Figures and Tables

**Table 1:** Total masses of the door and front barrel at different wall thicknesses.

Door Thickness (mm)	Front Barrel Thickness (mm)	Total Mass (g)
1.0	1.0	159.6
1.0	2.0	258.0
2.0	2.0	318.1
3.0	2.0	378.3
4.0	2.0	438.3
5.0	2.0	498.4
3.0	3.0	475.4
4.0	3.0	535.4

**Table 2:** Natural frequencies of 83.4 nm camera and filter assembly.

Position	Natural Frequency (Hz)
83.4 nm camera	56.3 Hz
Filter assembly	230.4 Hz

**Table 3:** Random vibration requirements.

Frequency (Hz)	Test Conditions	Acceleration (g_rms_, Root Mean Square)	Direction
10–95	+6 dB/oct	8.5	X/Y/Z
90–130	0.45 g^2^/Hz		
130–200	−14.7 dB/oct		
200–600	0.055 g^2^/Hz		
600–2000	−15 dB/oct		

**Table 4:** Acceleration responses under random vibration.

Position	Response to X Load (g_rms_)	Response to Y Load (g_rms_)	Response to Z Load (g_rms_)
83.4 nm camera	6.2	5.0	5.3
Filter assembly	10.4	6.1	5.9

**Table 5:** Acoustic test requirements.

Center Frequency (Hz)	SPL (dB)	Total SPL (dB)	Period (min)
31.5	118	142	1
63.0	123		
125	133		
250	135		
500	138		
1000	135		
2000	130		
4000	123		
8000	120		

**Table 6:** Random vibration test results.

Position	Response to X Load g_rms_	Response to Y Load g_rms_	Response to Z Load g_rms_
83.4 nm camera	7.4	4.4	4.4
Filter assembly	9.7	5.4	4.8

## Data Availability

The original contributions presented in this study are included in the article. Further inquiries can be directed to the corresponding author.

## Abbreviations

The following abbreviations are used in this manuscript:

EUV	Extreme ultraviolet
EUVC	Extreme ultraviolet camera
SPL	Sound pressure level
STL	Sound transmission loss
RMS	Root mean square
Al	Aluminum
Zr	Zirconium
In	Indium
